# Comparative study of environmental factors influencing motor task learning and memory retention in sighted and blind crayfish

**DOI:** 10.1002/brb3.106

**Published:** 2012-11-20

**Authors:** Sonya M Bierbower, Zhanna P Shuranova, Kert Viele, Robin L Cooper

**Affiliations:** 1 Department of Biology and Center for Muscle Biology, University of KentuckyLexington, Kentucky, 40506-0225, Russia; 2 Institute of Higher Nervous Activity and Neurophysiology, Russian Academy of SciencesMoscow, Russia; 3 Department of Statistics, University of KentuckyLexington, Kentucky, 40506-0027, Russia

**Keywords:** Cardiac, central nervous system, crustaceans, instrumental, respiratory

## Abstract

In classical conditioning, an alteration in response occurs when two stimuli are regularly paired in close succession. An area of particular research interest is classical conditioning with a chemical signal and visual and/or tactile stimuli as the unconditional stimuli, to test manipulative and motor behaviors in a learning paradigm. A classical learning task chamber was developed to examine learning trends in a sighted surface-dwelling crayfish, *Procambarus clarkii,* and in a blind cave-dwelling crayfish, *Orconectes australis packardi*. We examined whether learning is influenced by environmental factors and/or reliance on different primary sensory modalities. Crayfish were trained to manipulate a large, cumbersome cheliped through a small access point to obtain a food reward. In both species, acquisition of the learning task was rapid when they were in nonstressed conditions. The blind crayfish tested in low white light did not successfully complete the task, suggesting a stress response.

## Introduction

Researchers investigating associative learning in invertebrates have made significant breakthroughs in understanding the conditioning process in animals like *Aplysia* and honey bees (Couvillon and Bitterman 1980; Kandel and Schwartz 1982; Burmeitser et al. 1995). Studying invertebrate learning systems provides the opportunity to ask complex questions in relatively simple systems, as compared with vertebrates. An area of particular interest is the role of conditioning in learning through changes in behavior. Behavior is modulated by experience, through the acquisition of new information (learning) about the environment. Thus, instinctive behaviors can be modified based on the information provided in the environment.

Several invertebrate studies show that these organisms modify their behavior, especially avoidance behavior. This is seen in mollusks with habituation of the rapid gill withdrawal reflex (Castellucci and Kandel 1974), food aversion with electric shock (Mpitsos and Davis 1973; Mpitsos and Collins 1975), and CO_2_ poisoning (Gelperin 1975). One technique to demonstrate learning is using studies of operant learning, specifically the animal's ability to complete a task. A key study showed that *Carcinus maenas* (a crab) are able to perform a lever-press motor task (Abramson and Feinman 1990). Precise manipulation of appendages is a powerful behavior in learning abilities because it tests the degree to which manipulative and motor behaviors are part of paradigm motor command. This is especially interesting given our developing knowledge of neural circuitry and neuronal control in decapods such as crayfish and lobster (Krasne 1969; Davis 1970; Larimer et al. 1971).

Learning and memory formation are important in the natural environment and this is especially true for social animals, because many social hierarchies depend on recognition. As seen with many crustaceans, agonistic outcomes between conspecifics create a history of social experience that can influence future behavior (Goessmann et al. 2000; Daws et al. 2002; Bergman et al. 2003). Studies in mollusks have shown that they use sign or goal tracking (Kemenes and Benjamin 1989; Purdy et al. 1999). Although the exact mechanism has yet to be understood, learning and long-term memory formation are suggested to begin with long-term potentiation and maintained by prolonged strengthening of synapses to targets (Lynch 2004). Furthermore, many routine motor commands may use short-term plastic characteristics of neurons, as the neuromuscular junction in crustaceans shows short-term facilitation (Dudel and Kuffier 1961; Wiersma 1970). Thus, temporal codes formulated by common use pathways that may lead to more precise motor movements (Wilson and Davis 1965) are a possible explanation for the refinement of motor movements.

Freshwater crayfish provide a dramatic model of evolutionary adaptation in the contrast of sighted, *Procambarus clarkii* (surface) and blind, *Orconectes australis packardi* (cave) species. *Orconectes australis packardi* show typical cave-dwelling characteristics such as eye-structure modifications and reduced pigment (Mejia-Ortiz and Hartnoll 2005). They lack ommatidia and do not respond to visual cues (Cooper et al. 2001). Sighted crayfish have ommatidia and known visual capabilities both in and out of water. This provides an excellent model to examine whether similar species of crustaceans using different primary sensory modalities would differ in the rate of learning to complete a motor task. In this study, the multimodal integration of sensory input could be addressed by eliminating one particular sense with experimental manipulation or by altering the environment to examine what happens in a particular task when one modality is altered.

In this study, we examined learning in cave-adapted blind crayfish, in a novel setting, using a multitude of sensory modalities. The contributions of different senses to an organism's assessment of the environment create a complexity to the resulting learning, particularly with spatial orientation. In the paradigm we used, the crayfish had to spatially orient and complete a manipulation of a specific motor task, using both tactile and chemical sensory paths, to obtain a reward. If the learning of motor tasks is similar among different species with varied sensory modalities, the integrating centers that drive a learned motor command might be deciphered for anatomical and physiological identification.

This study investigates the use of an instinctive behavior to complete a learning task in a conditioning chamber. The task was for the crayfish to use one of their cumbersome chelipeds to reach into a hole only slightly larger than the cheliped itself to acquire a food reward. An unconditioned stimulus (chemosensory cue) with a conditioned response (access point to food reward) resulted in the reliable appearance of the response (manipulation of appendage). This is assumed to be driven by a chemical stimulus from the food itself.

The goals of the study were to (1) establish capability of crayfish to complete a motor task, (2) examine the impact of environmental influences on learning, and (3) determine if there are task learning differences between two species that rely on different primary sensory modalities. The hypothesis was that the cave crayfish would perform better than the sighted crayfish in an environment with red light creating hindered vision. To our knowledge, no other study of invertebrates examines whether environmental factors directly influence learning and task completion. Furthermore, no other study directly examines learning in cave crayfish.

## Materials and Methods

### Animals

*Procambarus clarkii* (sighted crayfish; 5.08- to 6.35-cm body length) were obtained commercially from Atchafalaya Biological Supply Co. (Raceland, LA). *Orconectes australis packardi* (blind crayfish; Rhoades; 4.5- to 6.35-cm body length) were obtained from Sloan's Valley Cave System (Somerset, KY; collecting permits obtained). These studies were conducted in Lexington, KY, between 2006 and 2009.

A total of 24 sighted and 24 blind crayfish were used. Both sexes were used, but learning differences between the sexes were not analyzed. Animals were housed individually in rectangular plastic containers and cared for in the same manner in an aquatic facility within our temperature-regulated laboratory (17–20°C). All animals were on a 12-h light–dark cycle, but *O. a. packardi* were covered with black plastic to omit light. They were fed dried fish pellets weekly until 2 weeks prior to experimentation. During experimentation, food was restricted to 30% of normal amounts. Because the crayfish were kept in a small container, their energetic needs were likely reduced. They were fed 1.2 g of “shrimp and plankton sticks: sinking mini sticks” (Aquadine, AquaDine Nutritional System, Healdsburg, CA). Crayfish handling was conducted using a glass beaker to transfer crayfish between containers. Because containers were cleaned weekly, the crayfish were handled often. This limited handling during experimentation is assumed to have little to no effect on the internal status of the crayfish. Only crayfish in the intermolt stage, possessing all walking legs and both chelipeds, were used.

### Chamber design

**Figure 1:**
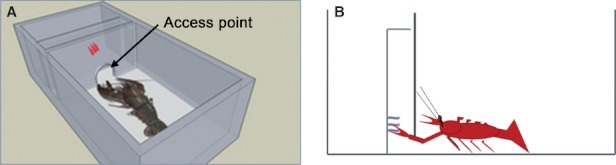
Schematic representation of the motor task conditioning chamber. The chamber is divided into two compartments, the larger one housing the animal and the smaller one containing a mesh platform with the food reward. Food was attached to the mesh screen. (A) A stylized angled view including the two compartments and mesh screen with worms attached. The location of the access point is indicated by the arrow. (B) Side view schematic to show placement of the mesh platform and the manipulative task of reaching in and up to obtain the food reward.

Four rectangular experimental chambers were constructed from Plexiglas (18 × 8 × 8 cm) with an 8-cm Plexiglas divider dividing one third of the container from the rest (Fig. 1). Sand was permanently glued to the bottom surface for traction. The crayfish were placed in the larger chamber. A vertical platform was placed in the smaller chamber, approximately 1 cm from the divider. The platform was a square plastic object (5.5 cm^2^) with mesh material on the surface. The access point was a half-oval shaped opening in the Plexiglas divider. This allowed only a single cheliped to enter into the smaller portion of the chamber (the hole was adjusted in size based on the species to be only slighter larger than a single cheliped). The food reward was five thawed bloodworms (mosquito larvae, PetCo, Lexington, KY) attached through the mesh material and placed into the chamber before the crayfish were added. The worms were centered 3 cm above the access point, which required the animal to reach in and up to obtain the food source. Each chamber was filled with carbon-filtered water to 2.54 cm from the top and aerated for at least 12 h prior to experimental trials. All four experimental chambers were simultaneously recorded by a digital video camera. Animals were placed in the chambers and each was secured with a Plexiglas lid. The animals were free to move within the chamber during the experiment. The Plexiglas was a common type obtained from a local hardware store (Home Depot, Lexington, KY).

### Experimental procedure and statistical analysis

**Figure 2:**
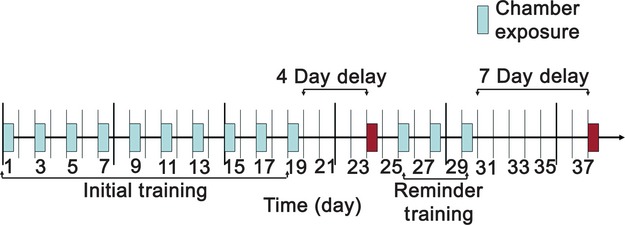
A graphical representation of the experimental training and testing. The light blue boxes represent exposure to the chamber and testing. The red boxes represent testing after a 4- or 7-day delay in exposure to the chamber.

A 3-week training period exposed all animals to the experimental chamber every other day starting at 08:00 between May and December. Each chamber exposure lasted until the crayfish pulled a single bloodworm from the mesh screen. There were four main studies: (1) low white light, 25 Lux (Lx), *P. clarkii*, *N* = 16; (2) red light 2.5 Lx, *P. clarkii*, *N* = 8; (3) low white light, 25 Lx, *O. a. packardi*, *N* = 8; (4) red light, 2.5 Lx, *O. a. packardi*, *N* = 16. After the training period, a 4-day delay was introduced to examine task retention. After this 4-day delay, all animals were placed into the chambers for 1 week of reminder training (one performed every other day for a total of four trials). Reminder training was used to ensure that all crayfish were at the same stage of learning before introducing the 7-day delay. Once the reminder training was completed, a 7-day delay was introduced. The conditioning trials were used to examine whether crayfish could learn a motor task. This paradigm also addressed if learning differences occurred between the two species. Ultimately, the comparison examined learning trends and whether visual sensory stimulation (sighted crayfish) aided in learning the motor task. We also examined if low white light had any effect on learning in blind crayfish. The 25 Lx illumination is a low-level mimicking periods of the day (dusk and dawn) when crayfish are known to be most active. Motor task learning was also examined in filtered red light (2.5 Lx) to remove the visual sensory system for the sighted crayfish. The red light (Kodak Adjustable Safeway Lamp, 15 W) allowed for video recording was previously noted to be a wavelength not detected by crayfish (Li et al. 2000; Li and Cooper 2002). During the time delay, these crayfish were not exposed to the experimental chamber and were housed in the same manner as all the other crayfish. A time line of the experimental conditions is shown in Figure 2.

All trials were digitally recorded and analyzed to record the time when the first worm was pulled from the mesh. The data collected were later analyzed with a timer and visual observation. Trial success was based on the removal of the first bloodworm. Quantification of learning was indexed from the time to pull the first worm on subsequent days. Thus, the data consist of raw data of individuals to complete the task and a mathematical formula to calculate a change over time per experimental group. To account for variability in individual rates of learning, each crayfish was analyzed for a percent change in learning over time. Raw data points are shown, as well as percent change values, which were determined by taking the absolute value of the first day of learning minus subsequent days, divided by the first day and multiplied by 100 to get a percent change from the first day of learning. The value is designated as a performance index (i.e., percent change from the first day). To understand trends, the values were averaged together to achieve an average percent change for each experiment. Quantification of memory is measured by the changes in task efficiency over repeated access to the experimental chamber after 4- or 7-day delays. Repeated measures analysis of variance (ANOVA) was performed using individual crayfish as random effects and day, species, and light condition as fixed effects. Post hoc analyses were conducted using a Bonferroni adjustment. When investigating the effect of day, pairwise comparisons were conducted only with respect to comparisons with Day 1 to measure learning effects (a significant decrease in task time from Day 1 indicates learning).

## Results

Two crayfish that did not perform the task on an experimental day were removed from subsequent trials and analysis.

**Figure 3:**
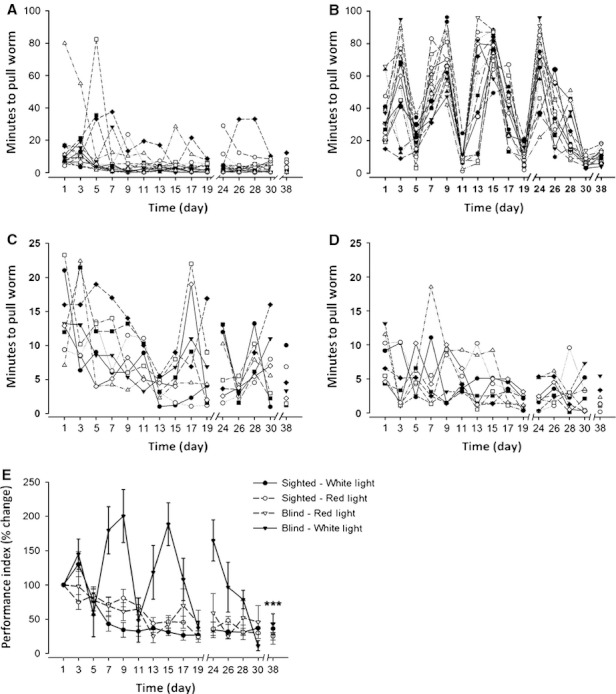
Graphical representation of species and environmental factor comparison in a motor task. Graphs show both sighted and blind crayfish in white and red light. Sighted (white light, *N* = 16; red light, *N* = 8) and blind crayfish (white light, *N* = 16; red light, *N* = 8). The experimental procedure consisted of chamber exposure every other day continually for 3 weeks followed by a delay of 4 and 7 days (indicated by breaks in *x*-axis). Raw data for individual crayfish in environmental conditions are shown in (A) sighted individuals in white light, (B) blind individuals in white light, (C) sighted individuals in red light, (D) blind individuals in red light, and (E) performance index was calculated as the change in time to complete the motor task from the first day of learning and averaged across each individual. The solid line represents white light and the dotted line represents red light. ***P < 0.001 for all comparisons.

The rate of learning varied among individuals in both initial task completion and task efficiency over time. To account for individual differences, we used the standard percent change formula (discussed in detail in the Methods section) for individuals and averaged across the group. For sighted crayfish in white light, the repeated measures ANOVA indicated a significant effect of Day (*F*_14,224_ = 3.53, *P* < 0.0001) with sizeable variation among the crayfish (residual standard deviation of 1.12 for log(Time) with a standard deviation across crayfish of 0.91). Thus, sighted crayfish in white light showed significant learning for all trials after 9 days (using a Bonferroni cutoff of *P* < 0.0035 to account for a family-wise error rate of *P* < 0.05). For blind crayfish with red light exposure, there was also a significant effect of Day (*F*_44,435_ = 3.83, *P* > 0.0001) with sizeable variation among crayfish (residual standard deviation of 1.42 for log(Time) with a standard deviation across crayfish of 0.96). For both sighted crayfish in white light and blind crayfish in red light, the actual length of time to pull the worm significantly decreased with each day after the ninth day (Fig 3). For sighted crayfish in red light, there was a significant increase in task efficiency over time (repeated measures ANOVA effect for Day had *F*_14,224_ = 3.26, *P* < 0.001; statistical significance not shown on graph). Post hoc comparisons using the Bonferroni adjustment for comparisons to Day 1 indicate that after 9 days, are all significantly faster than Day 1 (family-wise *P* < 0.02 after Bonferroni adjustments, individual *P* all ≤0.00115). The time to complete the task decreased from an average of 13 min to approximately 3 min overall (standard deviation among crayfish 4.55, estimated from the repeated measures ANOVA).

In contrast, blind crayfish in white light showed no such observed trend on a daily basis (only 1 day had a *t*-statistic less than [−2], which is not significant after accounting for the multiple comparisons). However, there was an overall learning difference between the first and last days of the experiment (*df* = 30, *t* = 3.78, *P* < 0.001; Fig. 3). Thus, blind crayfish in white light did not show a significant daily trend in increasing task efficiency due to the variation across days, but did show an overall decreased time to complete the task by the end of the experiment, to the point of not being significantly different from the other groups (Fig. 3).

Further detailed analysis examining only the environmental interference factor of white (visible) light versus red (invisible) light in the learning capability between the two species showed similar overall learning trends. Specifically, a statistical comparison of both sighted crayfish conditions (white and red light) to that of blind crayfish conditions (white and red light) showed no significant differences in overall learning between the two groups. The environmental factor of white light versus red light was investigated by fitting a repeated measures ANOVA that also included fixed terms for Light and the interaction of Light with Day (significance in the interaction term would indicate differing rates of learning). Using a backward elimination method, neither the interaction term nor the Light variable itself was significant for sighted and blind crayfish (*F*_14,224_ = 1.35, *P* = 0.18 for the interaction and *F*_1,224_ = 0.24, *P* = 0.62 for the main effect of Light). The performance index for blind crayfish in white light (Fig. 3) appears to oscillate, but there is no phased locked cycle that we could quantify.

**Figure 4:**
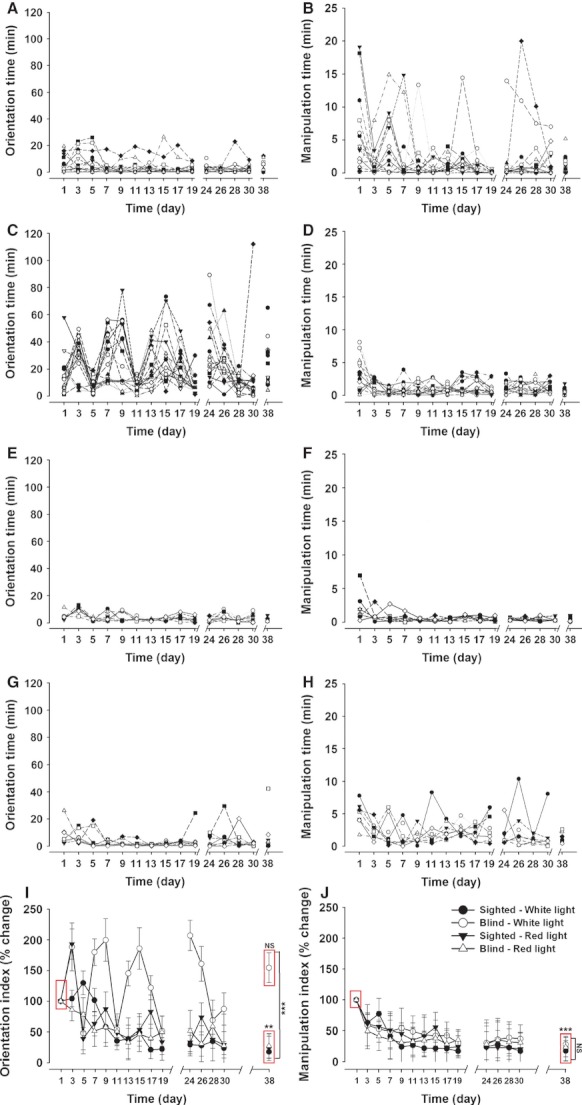
Graphical representation of orientation and manipulation times for both species in white and red light. (A) Sighted crayfish in white light showing individual minutes to orient and locate access point. (B) Sighted crayfish in white light showing individual minutes to manipulate the cheliped and remove reward. (C) Blind crayfish in white light showing individual minutes to orient and locate access point. (D) Blind crayfish in white light showing individual minutes to manipulate the cheliped and remove reward. (E) Sighted crayfish in red light showing individual minutes to orient and locate access point. (F) Sighted crayfish in red light showing individual minutes to manipulate the cheliped and remove reward. (G) Blind crayfish in red light showing individual minutes to orient and locate access point. (H) Blind crayfish in red light showing individual minutes to manipulate the cheliped and remove reward. (I) Orientation time only over the experiment. (J) Manipulation time. Orientation or manipulation index was calculated as the change in time from the first day of learning for each individual and then averaged across each group. Boxes indicate points of statistical comparison. ***P < 0.001 difference from Day 1. NS, no difference between groups on Day 38. The experimental procedure consisted of chamber exposure every other day continually for 3 weeks followed by a delay of 4 and 7 days (indicated by breaks in *x*-axis).

To understand the time difference between when the crayfish found the spatial access point and when they completed the motor task, further analysis of the performance index divided the total task time into orientation and manipulation index. The orientation index is defined as the time taken once the animal is placed in the chamber until it is in front of the access point (see Fig. 1A). If they approached the access point but did not attempt or complete the worm pulling, it was still considered orientation time. Manipulation index is defined as the duration after the animal starts to reach in through the access point until the time the food reward is pulled. Orientation time for sighted crayfish in both white and red light and blind crayfish in red light indicated a significant effect from the first day and last day of the experiment using the repeated measures ANOVA (*F*_4,79_ = 12.288, *P* < 0.001; using a Bonferroni cutoff of *P* < 0.0035 to account for a family-wise error rate of *P* < 0.05) with sizeable variation among the crayfish (residual standard deviation of 1.12 for log(Time) with the standard deviation across crayfish of 0.84; Fig. 4I).

In contrast, blind crayfish in white light did not show a significant difference from the first day to the last day of the experiment (*F*_4,79_ = 12.288, *P* < 0.028; using a Bonferroni cutoff of *P* < 0.0035 to account for a family-wise error rate of *P* < 0.05). Thus, there was a significant difference between the other experimental groups (sighted white/red light, blind red light) from Day 1 to Day 38, but not for blind crayfish in white light (Fig. 4I). Significance is shown on the graph with boxes representing the points of statistical comparison. Furthermore, group values on Day 38 were statistically tested across groups and showed no significance between sighted red/white light and blind red light, but there is a significant difference to blind white light (shown on graph).

To understand the actual time spent completing the motor task, analysis of manipulation index separated out the actual time between access point location and when the first worm was pulled. The manipulation index analysis indicated a significant effect for all experimental groups when comparing Day 1 and Day 38 (Fig. 4J).

Specifically, the repeated measures ANOVA comparison for sighted crayfish in white and red light, and blind crayfish in white and red light, showed a significant difference from the first day of learning (*F*_4,79_ = 5.78, *P* < 0.001) and no significant difference from each other with sizeable variation among the crayfish (residual standard deviation of 1.42 for log(Time) with the standard deviation across crayfish of 0.93). Significance is shown on the graph with boxes representing the points of statistical comparison (Day 1, Day 38) as well as significance between groups on Day 38. Sighted crayfish showed more individual variability during manipulation time with white light, so the ability to see the food reward may have interfered with the ability to manipulate the cheliped in the same time frame that occurred in red light.

In comparing orientation and manipulation time, it becomes apparent that blind cave crayfish in white light wander and do not find the access point as quickly as they do in the dark or as well as the sighted crayfish. This is indicated by a nonsignificant effect from the initial start of the trial and high variability each day, which was not seen with any of the other experimental groups (Fig. 4C). The orientation time shows a cyclic-like pattern for blind crayfish in white light (Fig. 4I), but it is not consistent or phased locked.

When manipulation time was separated out, the blind crayfish in white light completed the motor task and had the same learning trend as blind crayfish in red light and sighted crayfish in both white and red light (Fig. 4B, D, F, and H). Thus, for manipulation time alone, the actual length of time to pull the worm significantly decreased with each day for all groups (Fig. 4J).

## Discussion

In this study, we compared learning trends in sighted and blind crayfish and provided the first study on blind cave crayfish learning. Specifically, we examined classical conditioning in which the chemical signal is the unconditional stimulus and the access point is the conditional stimulus; thus, the reach from the crayfish and food reward becomes the unconditional response. In this study, we quantified: (1) the ability to complete a motor task, (2) how rapid the acquisition occurred, (3) how efficient the performance was, and (4) how well the animals retained the learned task. We established that crayfish have the ability to use an instinctive behavior to learn and complete a specific motor task. To complete a motor task, sighted crayfish could be assumed to rely heavily on visual and chemosensory cues for task efficiency. Yet, when visual sensory information was removed, we found that visual cues were not required for task completion. This was similar to that the situation in blind crayfish, which rely on tactile and chemosensory modalities instead of visual sensory information. For some crabs and crayfish, chemosensory responses are known to occur when chelipeds alone are exposed to chemical cues (Holmes and Homuth 1910; Hartman and Hartman 1977). However, much of the behavioral exploration of *P. clarkii* has been observed to rely heavily on visual cues.

We suggest that a learning trend occurred in *P. clarkii* with reliance on various primary sensory modalities. Furthermore, environmental influences may impact learning by inducing a stress response. Interestingly, the sighted crayfish quickly learned to complete the task (5–7 days) which suggests they easily habituated to the task chamber. This behavioral task is indicative of a behavior possibly used in the natural environment. Although sighted crayfish are known to rely on visual sensory information about the environment (Bruski and Dunham 1987; Smith and Dunham 1990), they also use sensory integration of tactile and olfactory cues for behavioral responses (Bovbjerg 1953, 1956; Rutherford et al. 1996; Issa et al. 1999; Zulandt-Schneider et al. 1999; Goessmann et al. 2000). Because the performance index has orientation time as a subset of the measure, it might be expected that the slight oscillatory effect is seen in both measures (Figs. 3, 4B and I). The oscillatory effect is also observed in the separate trial components of orientation and manipulation during the behavioral trials. This is illustrated for the blind crayfish in white light as individuals (Fig. 4C) and in the composite data (Fig. 4I). The mechanism for this “cyclic-like” behavior is not known. It is interesting that it occurs for the cave crayfish exposed to white light. Possible mechanisms include a stress hormone or receptor expression cycle due to the continuous light stress when exposed to the task chambers. When the cave crayfish were not being tested they were held in the dark. It may be that a different pattern would be observed if they were held continuously in white light, even between test trials.

It is possible that this experimental motor task is not true motor learning (i.e., development of a motor habit) but is only an increase in approach of the food source. However, analyses which divided orientation time from manipulation time demonstrated that both species of crayfish approached the access point faster and improved their cheliped manipulation skills. This increased task efficiency over time indicates a learned motor task. A decrease in the latency to take the worm over time suggests that the animal is learning how to manipulate the cheliped into the small space and rotating the cheliped up to reach the food. This manipulation is the motor task measured. In addition, when examining individual crayfish over time for each trial, the animals (both blind and sighted) did not show a preference for one cheliped over the other, nor did they show a preference throughout the repeated trials. Perhaps, if the blood worm was placed more to one side of the screen, the animals would have only been able to reach it with one cheliped and we could have examined if the repeated trials showed an initial preference for the left or right cheliped. This would make an interesting future investigation.

To our knowledge, this is the first study to address cave crayfish learning. It would be of interest to compare the neural architecture between these two species of crayfish. If regions within the central brain were more readily accessible for ablation in the intact animal, or if crustaceans were amenable to genetic manipulations of particular neurons, as for *Drosophila*, one could gain further insight in the functioning of the higher centers of crustaceans. Perhaps, approaches with RNA interface might allow targeted actions if specific mRNAs could be identified for known neuronal types (Pekhletsky et al. 1996; Mario et al. 2007; Kato et al. 2011). In most crustacean species, the regions of the nervous system responsible for learning are not well known. However, it is known that in crayfish and lobsters, tactile, chemosensory, and visual information all project to the central brain (see Sandeman et al. 1992; Mellon 2000; Cooper et al. 2001). To understand the complexity of investigating learning in crustaceans, a crustacean that lacks visual sensory structures reduces the complexity of the integration with other senses, thus narrowing the focus of which senses can drive learning and memory in crayfish. Past studies of operant learning in Crustacea have been simple position habits (i.e., Y- or T-mazes in Tierney and Lee 2011; eye withdrawal in Abramson et al., Abramson and Feinman 1988; lever-press in Abramson and Feinman 1990; Tomina and Takahata 2010, 2012) or punishment schemes developed by Horridge (Yerkes and Huggins 1903; Schwartz and Safir 1915; Gilhousen 1929; Datta et al. 1960; Schone 1961; Horridge 1962; Harless 1967; Abramson and Feinman 1987; McMahon et al. 2005). The findings of our study demonstrate that environmental factors which can induce a stress response significantly impact learning and provide a foundation for reasons behind complex behaviors.

Future investigations can be directed to determine the regions of the crayfish central nervous system responsible for learning, like the mushroom bodies in *Drosophila* (De Belle and Heisenberg 1994) or the cerebellum, as suggested for mammals in motor learning (Eccles et al. 1967; Marr 1969; Ito 2006). It would also be interesting to understand the cellular mechanisms for motor task learning.

## Conclusion

In summary, we demonstrated the ability of crayfish to learn and remember a location and a motor task. We also demonstrated that there was no difference in learning between two species of crayfish that rely on different primary sensory modalities. However, learning was impacted when blind crayfish were exposed to low white light, as indicated by the increased time spent in the orientation phase of the trials. Activating the caudal photoreceptor may induce a stress response not observed in the absence of light.
